# Does Self-Efficacy Affect Clinical Reasoning in Dental Students?

**DOI:** 10.1016/j.identj.2022.05.006

**Published:** 2022-06-23

**Authors:** Ebtihaj T. Nafea

**Affiliations:** Department of Dental Education, Taibah University, Medinah, Saudi Arabia

**Keywords:** Academic achievement, Clinical reasoning, Dental education, Dental students, Self-efficacy

## Abstract

**Introduction:**

Individual ability, motivation, age, and gender are all specific learner-related factors that can affect the academic performance of undergraduate dental students. Clinical reasoning and self-efficacy may potentially play crucial roles in this. This research aims to study the effects of clinical reasoning and self-efficacy on academic performance and to determine the relationship between them.

**Materials and methods:**

This is a cross-sectional quantitative study that was conducted in 2021, and the study participants included 81 (86.19% response rate) final-year dental students who responded to an online questionnaire containing a specially designed clinical reasoning test and a self-efficacy scale.

**Results:**

Although the levels of clinical reasoning skills and self-efficacy were not directly related, they were positively associated with students’ academic achievements. Furthermore, these 2 factors were considered to be predictors of a student's academic performance.

**Conclusions:**

High levels of self-efficacy and clinical reasoning skills were associated with high academic achievement in students. However, having high levels of self-efficacy does not necessarily indicate mastery of clinical reasoning skills. This conclusion reflects the complexity of the clinical reasoning process during which an individual is faced with uncertainty. High levels of confidence might make students rush to a conclusion without considering all the conflicting possibilities or alternatives. In all instances, dental educators should follow recommended measures to improve clinical reasoning and self-efficacy abilities due to their importance in improving learning in students.

## Introduction

Educational programmes are usually evaluated based on the outcomes of the teaching and learning processes that occur at a specific institution. Students’ academic performance is one such important measurable outcome.^1^ Identifying factors that influence students’ academic performance is critical for enhancing teaching programmes, and this has been a popular area of research in health professions education.

One of the most important targets of undergraduate dental education, and all other health profession education, is to impart high levels of clinical reasoning amongst students.^2^ Clinical reasoning skills are considered the most important of all skills.^3^, ^4^, ^5^ They allow health professionals to use various cognitive approaches to determine the best diagnosis and formulate the most suitable treatment plans in an interactive phenomenon. During this process, the health professional works through each case and practices specific skills, such as gathering information, synthesising hypotheses, and conducting tests. This process is better described as an interactive phenomenon rather than being referred to as clinical judgment, critical thinking, decision-making, and problem-solving, which is the usual practice in the medical literature.^2^^,^^6^, ^7^, ^8^ The interactive nature of clinical reasoning can be attributed to the fact that health professionals handle many emergent situations related to patients, themselves, or various contexts.^8^ However, despite its importance, clinical reasoning is still under-researched in the field of dentistry.^7^ Evaluation of students’ performance usually involves assessment of the skills that compose clinical reasoning. There are many assessment tools described in the medical literature that are devoted to testing clinical reasoning skills and can be applied to dentistry. Some of these tools aim at assessing the end product of clinical reasoning, whilst others assess the process itself.^8^ Combining more than one assessment tool might boost the efficacy of the assessment process.^8^, ^9^, ^10^

Self-efficacy is another factor that has been proven to be related to the academic performance, motivation, and learning outcomes of undergraduate health care students.^11^, ^12^, ^13^, ^14^ This term refers to individuals’ perceptions of their own competency regarding the performance of a skill or task.^15^ It has been suggested that individuals’ levels of self-efficacy affect their performance of tasks. Therefore, it is important to manipulate these levels to influence and modify the outcomes of the action(s) taken.^16^ Furthermore, becoming more confident in certain skills can also empower students to handle complex and stressful situations more effectively, which is an important quality when treating patients.^8^^,^^17^ These skills are crucial during the process of clinical reasoning. Confidence also affects medical students’ career development, stress levels, and problem-solving abilities.^18^ On the contrary, low self-efficacy levels were also found to be associated with increased academic stress and academic burnout in graduate students.^19^ Thus, both clinical reasoning and self-efficacy are considered important factors affecting the students’ academic performance. However, no research was found in the dental literature that either discusses the association between clinical reasoning and self-efficacy or discovers their possible combined effect on students’ academic performance. Furthermore, dental literature is still deficient in exploring additional factors that might affect clinical reasoning despite its paramount importance. In the current study, a special innovative tool was used to comprehensively assess clinical reasoning skills and study the association amongst 3 independent factors (academic performance, clinical reasoning, and self-efficacy).

The objectives of this study were as follows:

• Examine the relationship between clinical reasoning skill levels and academic performance in dental students.
• Explore the effect of dental students’ self-efficacy on their academic performance.
• Determine the relationship amongst academic performance, clinical reasoning, and self-efficacy.

## Materials and methods

This cross-sectional quantitative study was approved by Taibah University, College of Dentistry Research Ethics on September 8, 2020. Its targets were final-year undergraduate dental students enrolled in the BDS (Bachelor of Dental Surgery) programme at Taibah University. The BDS programme takes 6 years to complete and follows the semester system with a traditional 2-phase curriculum. The preclinical phase lasts 3 years, during which basic biomedical science knowledge is taught in traditional lecture-based education. Then students proceed to the clinical phase for another 3 years, during which clinical and paraclinical subjects are conducted at the college hospital and seminar rooms. After finishing their sixth year, students are enrolled in a compulsory rotatory internship programme carried out at the college hospital and outreach dental clinics for an additional year. Sixth-year students are required to finish at least one complete clinical case with minimal clinical practice requirements in each specialty/discipline at the university hospital.

At the time of conducting the current study, there were 2 batches of sixth-year students. This was due to the lockdown caused by the COVID-19 pandemic. The total number of senior students in the older batch was 49 (22 men and 27 women). Some of the students in this batch were still working on their comprehensive clinical cases during the extra evening sessions. However, they had finished their taught courses, including lectures and seminars. On the other hand, the current group of junior students had a total of 45 (24 men and 21 women) students. The current study was carried out 1 month before the conclusion of their final year. Both senior and junior groups were included as they met the inclusion criteria of the study, being final-year undergraduate dental students who had finished or nearly finished their taught courses, including lectures and seminars. Other students who did not meet the inclusion criteria were excluded from the current study. Sixth-year students were targeted as participants to ensure that almost all the students in the sample would have attained the maximum level of basic biomedical science knowledge and practical training required to develop their clinical reasoning skills and facilitate the study analysis.

An online questionnaire was used to collect data, which were then shared via Google Forms. The questionnaire comprised 3 parts: demographic data in addition to a question on academic achievement measured by the *grade point average (GPA)*, a specially designed *clinical reasoning test (CRT)*, and the *General Self-Efficacy Scale (GSS)*. The questionnaire was presented in English as it was the language of instruction in their course. Please refer to Appendix 1 for the questionnaire.

An online link was emailed to the participants along with 2 reminder emails, and they were encouraged to participate in a raffle for online gift vouchers. Before answering the questions, the respondents were informed that their participation in the questionnaire survey was voluntary and anonymous. Data collection was conducted over 2 months beginning in January 2021. The collected data were split into 3 variables.

For the first part of the questionnaire survey (GPA), participants were asked to choose their accumulative GPA from the provided list, including both clinical and nonclinical grades as presented in their academic transcripts. Using GPA as an indication of academic performance in the current study was suggested based on the assumption that mastering basic academic knowledge (taught in preclinical and paraclinical subjects) together with practical experience constitutes important parts of clinical reasoning.^6^ This part was scored as follows: A+ = 9, A = 8, B+ = 7, B = 6, C+ = 5, C = 4, D+ = 3, D = 2, F = 1. The maximum score for this section of the questionnaire was 9 and the minimum was 1.

The CRT was composed of 31 items intentionally developed in an innovative manner using a combination of assessment tools (including a modified version of the Key Feature items,^20^ Patient Management Problems,^21^ Script Concordance Test,^22^ and a few questions testing participants’ biomedical science knowledge^23^). Using more than one assessment tool was suggested to test the various elements of clinical reasoning in a single test. The same test was previously applied in a separate research.^8^^,^^24^ Its content, construct and face validities, and suitability for final-year dental students were examined and approved.^8^^,^^24^ The CRT results were scored both manually by a single evaluator for short text answers (looking for key words) and electronically for multiple choice and multiple answers. The total possible score on the CRT was 45. Please refer to Appendix 2 for more details on the CRT marking.

The last part of the questionnaire comprised the GSS, which consists of 10 items. This scale is the most commonly used self-reported tool to measure one's self-efficacy level. It was originally developed by Schwarzer and Jerusalem in 1992.^25^ The scale has been widely used in medical and dental literature; it has been used in more than 1000 studies across many countries and in various languages, with high levels of validity and reliability.^26^^,^^27^ It is composed of a 4-point rating scale to assess the perception levels of the participants, which were scored as follows: 1 = not at all true, 2 = hardly true, 3 = moderately true, and 4 = exactly true. The scores for this part ranged from 10 to 40.

The data were analysed using SPSS version 26 (IBM Corporation). Cronbach's alpha was calculated to measure the reliability of the questionnaire items. Normality tests were conducted for the data using numerical and visual outputs. A statistical comparison of means using nonparametric tests was conducted. The 3 independent variables in the questionnaire were examined using a 3-way analysis of variance (ANOVA), a pairwise comparison test, and the Kruskal–Wallis test for the paired samples with Bonferroni correction. *P* < .05 was considered statistically significant for all the tests. Correlation coefficients and modified linear regression were also assessed for possible associations amongst GPA, CRT, and GSS results. The significance level was set at 5%.

## Results

The reliability value of the CRT items (indicated by Cronbach's alpha) was 0.899, and it was 0.657 for the GSS items. The questionnaire's combined internal reliability was acceptable (*α* = 0.778). Eighty-one out of 94 senior and junior students participated in the current study, including 41 women and 40 men. The response rate among the 2 batches was 85.42% for the seniors and 86.96% for the juniors. Normality tests indicated that some of the data were not normally distributed (Shapiro–Wilk values for CRT scores for the men and women were 0.238 and 0.009, respectively, and the GSS scores for the men and women were 0.001 and 0.209, respectively). The nonnormal distribution was also reflected in their histograms. Therefore, nonparametric tests were used in the analysis.

The Mann–Whitney U test was used to compare the mean scores of GPA, GSS, and CRT obtained by the 2 groups. None of the *P* values reflected any difference between the senior and junior groups of students (Table 1). It was therefore decided to combine their results into a single group. The detailed mean values for the male and female samples are presented in Table 2. The results obtained from the Kruskal–Wallis test revealed that there was no statistical difference between the mean scores obtained by the participants of different genders with regard to their CRT and GSS, *P* = .060 and .758, respectively. However, female students had significantly higher GPAs than male students (*P* = 0.00).

**Table 1:** Mann–Whitney U test indicating no statistical difference (*P* > .005) between the junior and senior groups in their total clinical reasoning test (CRT), General Self-Efficacy Scale (GSS), and grade point average (GPA) scores.

Comparison between the seniors and juniors	*P* value
Total CRT scores	.557
Total GSS scores	.587
Total GPA scores	.451

**Table 2:** Mean and standard deviation values for grade point average (GPA), total clinical reasoning test (CRT), and General Self-Efficacy Scale (GSS) scores obtained by the male and female participants.

Gender		GPA	CRT score	GSS score
Male	N	40	40	40
	Mean (SD)	6.08 (1.44)	23.89 (5.01)	27.75 (3.61)
Female	N	41	41	41
	Mean (SD)	7.71(1.93)	25.86 (7.31)	28.07(4.13)
Total	N	81	81	81
	Mean (SD)	6.90 (1.88)	24.89 (6.32)	27.91 (3.86)

Correlations amongst the 3 variables were studied for the two genders separately. The results indicated that there were no significant correlations between the CRT and GSS scores for the male and female participants (*R* = 0.108 and *R* = 0.101, respectively). However, positive correlations were found between GPA and RCT for the men and women (*R* = 0.556 and *R* = 0.610, respectively). Furthermore, positive correlations were found between GPA and GSS for the male and female samples (*R* = 0.306 and *R* = 0.386, respectively).

Correlations amongst the 3 variables were also studied for the whole sample (men and women). Similarly, the results indicated that there was no correlation between the CRT and GSS scores (*R* = 0.152). A moderate positive association was found between GPA and CRT (*R* = 0.476), and a low positive correlation was found between the GPA and GSS results (*R* = 0.301; Table 3).

**Table 3:** Summary of the relationships amongst the study variables (clinical reasoning test [CRT], grade point average [GPA], and general self-efficacy scale [GSS] scores) for the whole sample.

Variables	Pearson correlation	Level of correlation	Regression	Conclusion
CRT as a dependent variable on GPA	0.476	Moderate positive	*R* value (0.476), *F* (23.196), *P* for ANOVA (.001)	CRT score is a predictor for GPA
GSS as a dependent variable on GPA	0.301	Low positive	*R* value (0.301), *F* (7.895), *P* for ANOVA (.006)	GSS is a predictor for GPA
GSS and CRT	0.152	No correlation	There was no significant correlation	No relationship between GSS and CRT scores

ANOVA, analysis of variance.

Curve fitting was performed (Figure), and the modified multiple linear regression analyses revealed that the CRT score was a predictor for GPA (*R* = 0.476 > 0.4, *F* = 23.196, and *P* value of ANOVA = .001 < .05). Furthermore, the GSS score was also a predictor for GPA (*R* = 0.301, *F* = 7.895, *P* value of ANOVA = .006).

**Fig:**
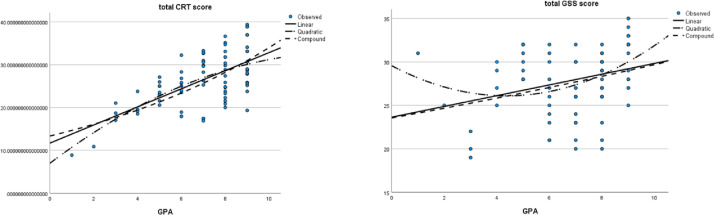
Curve fitting diagram for the 3 variables, grade point average (GPA), total clinical reasoning test (CRT) score, and total General Self-Efficacy Scale (GSS) score for the whole sample.

## Discussion

Studying the factors affecting the students’ academic performance is important to ensure a good teaching and learning experience. To our knowledge, the current study is the first to evaluate the relationship amongst the 3 important variables in undergraduate dental education: clinical reasoning, self-efficacy, and academic achievement. This relationship was analysed after the clinical reasoning skills were thoroughly assessed, using a specially designed and validated tool (CRT). In addition to the CRT, the questionnaire used in this study has another part measuring the self-efficacy level of the participants (GSS). Scores from both parts were then studied together with the GPA.

The results of the current study indicated that both the senior and junior groups of students performed similarly in answering the questionnaire, with no statistical difference between them regarding their GPA, CRT, and GSS scores. This finding might indicate that both groups had attained the required knowledge and skills to graduate in a similar way without the possible negative effects of the pandemic lockdown. Similar results were found in the dental literature regarding the effect of the pandemic curriculum modification on the performance of dental students who performed even better than their predecessors.^28^

The gender effect on students’ academic performance was also studied in the current research. Although the findings indicated that female students had significantly higher GPAs than males, as has been suggested in dental literature,^29^ their clinical reasoning skills were not affected by gender. Similar findings highlighting no gender effect on clinical reasoning skills have been reported in the medical and dental literature.^8^^,^^10^^,^^30^^,^^31^ In addition, there was no difference in the self-efficacy scores between male and female students. This finding has been supported by similar findings in other studies in the literature, including those conducted by the GSS creator.^11^^,^^25^ However, the results of the current study contradict the results of a study which found that final-year male dental students to have higher self-efficacy scores than final-year female dental students.^32^

According to Albert Bandura's social cognitive theory, the beliefs that individuals have in their ability are fundamental to their actions, the mediation between knowledge and application and, hence, their behaviour.^33^ For that reason, self-efficacy is considered a very important factor that could manipulate the thoughts and actions of students and, consequently, their academic performance. The results of the current study suggest that higher levels of self-efficacy were found to be associated with higher GPA (*P* = 0.30). They also indicated that self-efficacy is a predictor for high academic performance, *R* = 0.476, *F* = 23.196, *P* for ANOVA = 0.001. Similar results have been reported in other studies in the dental literature.^34^, ^35^, ^36^, ^37^ The importance of self-efficacy has also been highlighted in medical literature, with students reporting that confidence had a calming effect and led to a better self-perceived performance.^38^ Furthermore, self-efficacy is perceived to be a predictor of students’ learning and motivation.^39^

The results of the current study also indicated that higher levels of clinical reasoning were found to be associated with higher academic achievement (*P* = 0.476). Furthermore, clinical reasoning was found to be a predictor for higher academic achievement (*R* = 0.476, *F* = 23.196, *P* for ANOVA = .001). The results of the current research and the previously mentioned studies highlight the importance of self-efficacy and clinical reasoning in improving students’ academic performance. In response to the findings, the author suggests a possible positive relationship between self-efficacy and clinical reasoning, as both are associated with higher academic performance. This suggestion was made because one can easily infer that a higher level of self-efficacy might boost an individual's ability to take the actions required in the process of clinical reasoning. However, as far as I know, this relationship, in particular, has not been investigated, either in the medical or dental literature. On the other hand, there was research conducted in the education literature^40^ with findings that supported the association between self-efficacy and the problem-solving ability of the learner, as belief in one's self-efficacy influences individuals’ thought patterns and emotional reactions. People with low self-efficacy levels believe that things are tougher than they are. This leads to stress and feelings of depression, in addition to limiting their problem-solving abilities. Therefore, assessing and improving self-efficacy beliefs may help provide students with emotional and social support.^40^ Although the factor discussed in the previously mentioned study was problem-solving ability rather than clinical reasoning, we can still relate the results to clinical reasoning. The reasons behind this suggestion were based on the fact that we can consider problem-solving as part of the reasoning process.^6^^,^^8^ As described earlier, the interactive process of clinical reasoning involves many skills and tasks, including the gathering of important information, the selection of diagnostic tests, decision-making, and problem-solving. The current research used a valid and intentionally created clinical reasoning test that is claimed to assess almost all the components of the clinical reasoning process, combining the advantages of multiple well-known assessment tools.

However, the findings of the current study suggested that there was no correlation between self-efficacy and clinical reasoning scores (*P* = 0.152). Nevertheless, each of these scores was positively associated with higher academic performance. The absence of a positive relationship between these factors might further stress the complexity of the interactive process of clinical reasoning, which cannot be simply reflected or explained by the concepts of self-efficacy. Self-efficacy levels might affect how fast and confident the student is, but not necessarily whether they reach the correct end product of the reasoning process. Students, on the contrary, should think about and doubt their ideas to go through the process of clinical reasoning. During this process, they need to be more flexible in thinking and considering multiple thoughts and think about all alternatives and possibilities rather than confidently rushing to a conclusion. Further research is needed to study this effect.

Highlighting the positive effect of high self-efficacy in dental education, recommendations from dental literature state that specific strategies may increase levels of self-efficacy, such as the number of supervised clinical sessions and rotation as well as the number of successful treatments performed.^26^^,^^27^^,^^41^^,^^42^ Furthermore, the application of integrated teaching strategies was also associated with higher levels of self-efficacy.^43^ Other recommendations regarding clinical reasoning were found in the dental literature. It was found that exposing dental students to as many clinical cases as possible was considered an important strategy in improving their clinical reasoning ability.^7^^,^^8^ This recommendation was made based on the fact that pattern recognition, in particular, is the most important type of clinical reasoning in dental students. During this specific type of clinical reasoning, students relate information from the current patient's case to a previous similar case and work in a deductive manner to arrive at a possible diagnosis. This type was mostly associated with correct diagnoses.^7^^,^^8^

## Conclusions

This study highlighted the importance of self-efficacy and clinical reasoning abilities as they were found to be predictors of higher academic achievement in students, despite that they were statistically unrelated to each other. Being unrelated to each other does not mean that self-efficacy has no effect on clinical reasoning. More research can be conducted to study the possibility of these effects on the components or stages of clinical reasoning. In conclusion, dental schools should follow recommendations to enhance students’ self-efficacy and consider assessing its level for their students as well as implementing services to help those students demonstrating low levels. They should also cultivate the development of clinical reasoning skills throughout the learning process.

There was a possibility of reporting bias in this study as it relied partly on a self-reported survey for the GPA and GSS sections. The findings may not be generalisable, and more research is needed to study the factors affecting self-efficacy and the effectiveness of helping programmes. There is evidence in the literature to suggest that the prediction of academic performance is based on cognitive capacity, assessment of past achievement, individual differences, and other non-intellective factors.^44^ Therefore, using the GPA (a form of assessing academic performance) as a single predictor to judge clinical reasoning and self-efficacy does not seem to cover all possible factors.

## Conflict of interest

None disclosed.

## Appendix Supplementary materials

Image, application 1

Image, application 2

Image, application 3

Image, application 4

## Footnotes

Supplementary material associated with this article can be found in the online version at doi:10.1016/j.identj.2022.05.006.
