# Supplementary material for: Does Self-Efficacy Affect Clinical Reasoning in Dental Students?
Source: Int Dent J. 2022 Jun 23;72(6):872–8. doi: 10.1016/j.identj.2022.05.006 (PMC9676534; doi:10.1016/j.identj.2022.05.006)
Supplement: Supplementary file 1 [file mmc1.pdf]

# Clinical Reasoning Test

Dear sixth year students,
I am carrying out a study which tends to examine the relationship between clinical reasoning skills, self-efficacy and academic performance. Because you are a sixth year dental student, I am inviting you to participate in this research study by completing the following form.
This study will provide important information and guidelines to dental educators and course planners to improve clinical reasoning skills within the undergraduate dental curriculum.
I would be very pleased if you could spare time to take part in my research. The following questionnaire will require 15 to 20 minutes to complete. Please note that for the purpose of this research it is not always possible to see the previous screen once you have completed it.
Please be assured that all information collected during this research will be kept strictly anonymous and confidential. A summary of the findings of this study will be forwarded to you upon request. In addition, participation is voluntary and you may refuse to participate at any time. You will have the oppertunity to enter a raffle to win YouGotaGift® vouchers as a thank you for your effort.
Thank you for taking time to assist my educational endeavors. If you have questions or concerns please do not hesitate to contact me at: ebtihajnafea@gmail.com

* Required

1. Which group are you from? *

*Mark only one oval.*

- ◯ 2020 (DENT 35)
- ◯ 2021 graduates (DENT 36)

2. Gender *

*Mark only one oval.*

- ◯ Female
- ◯ Male

3. What is your General grade point? *

*Mark only one oval.*

- 4-5 (A+)
- 3.75-4.75 (A)
- 3.5-4.5 (B+)
- 3-4 (B)
- 2.5-3.5 (C+)
- 2-3 (C)
- 1.5-2.5 (D+)
- 1-2 (D)
- 0-1 (F)

4. Q1. List the features from the case description below that could help you in making a diagnosis (List at least three) *

## Case 1

A 30-year-old lady came to your dental office complaining of pain related to her lower left molars, especially when biting. She reported the pain as moderate.

Past medical and dental history

The patient is medically fit and is not on any medication. She reported that she is used to having regular visits to the dentist. Her last visit to the dentist was about 3 weeks ago.

Extra-oral examination

No abnormality observed.

Intra-oral examination

Fairly good oral hygiene was noticed with healthy gingival tissue. She has class I occlusion with cross bite of her canines on both sides. Class I occlusal composite restorations to her teeth: UR7, UL6, LL7 and LR7. The UR6 has a class I amalgam filling. She also has fissure sealant for her premolars. The LL7, with an adequate occlusal class I composite, was sensitive to vertical percussion. Generalised attrition to occlusal surfaces was observed. By asking the patient, she confirmed that she often clenches her teeth especially when stressed.

Radiographic examination

No periapical lesion was noticed in the area of concern.

5. Q2: At this stage, What is your most likely diagnosis for the LL7? *

6. Q3: What further investigations will help to diagnose this problem, or confirm your diagnosis?

Untitled Section

7. Q4. If you suppose that this lady has a high composite filling for LL7, and by reviewing her dental record, it was found that her dentist had used composite designed for the use in anterior teeth, how can you manage her problem? Choose the best answer. *

*Check all that apply.*

- [ ] Slightly reduce the filling by 0.5mm.
- [ ] Replace the filling by a new one with composite for posterior teeth
- [ ] Do a night guard (splint)
- [ ] Ignore the problem as it will go away with time, it will undergo attrition by clenching
- [ ] Identify high points and reduce the filling
- [ ] Refer her to a restorative dentist
- [ ] Replace the filling with amalgam

8. Q5: How could this problem be avoided in the first place?

Case 2

## Case2

A 16-year-old male came to your clinic complaining of brownish discolouration and irregular teeth related to his anterior teeth as seen in the picture. He is shy and does not want to show his teeth. He also stated that he's had bad teeth since he was a child but he's now started to take care of them and they are improving. His nationality is Indian and he came to live in the UK 8 years ago.

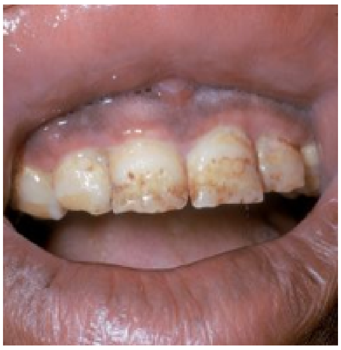

9. Q6. What clinical features from the above description would you focus on in making your diagnosis for this patient? List up to 3

10. Q7. If you could choose the course of action to follow, what would you like to do immediately? (choose one) *

*Mark only one oval.*

- I don’t know
- Ask the patient more questions
- Conduct intraoral examination
- Give him oral hygiene instructions and do scaling.
- Take an x-ray
- Refer the patient to an oral pathology specialist

11. Q8. From the list below select the three most important questions that you would like to ask the patient. *

*Check all that apply.*

- [ ] • Do you have other siblings affected?
- [ ] • How frequently do you brush your teeth?
- [ ] • Do you use fluoridated tooth paste?
- [ ] • Do you have any pain or sensitivity?
- [ ] • Have you experienced trauma to your teeth?
- [ ] • Do you drink lots of coffee or tea?
- [ ] • What type of water you used to drink when you were younger?
- [ ] • Did you have any serious illness when you were a baby?
- [ ] . Do you use Chlorhexidine mouth wash?

the patient's answer to previous questions

• Do you have other siblings affected? I have a 4-year old sister and she hasn’t got this problem
• How frequently do you brush your teeth? I brush my teeth twice a day
• Do you use fluoridated tooth paste? Yes
• Do you have any pain or sensitivity? No
• Have you experienced trauma to your teeth? Can’t remember
• Do you drink lots of coffee or tea? I usually take two cups of black coffee daily
• What type of water you used to drink when you were younger? Tap water
• Did you have any serious illness when you were a baby? No, I don’t think so
. Do you use Chlorhexidine mouth wash? Yes I usually use it once a day for about three months

12. Q9. Select three results from the list below that you would expect to find during examination *

*Check all that apply.*

- [ ] Generalized discoloration
- [ ] Caries
- [ ] Generalized recession
- [ ] Localized discoloration
- [ ] Open bite
- [ ] Small teeth
- [ ] Loss of proximal contact
- [ ] Abrasion and horizontal bone loss
- [ ] Vertical bone loss
- [ ] Submandibular gland tenderness
- [ ] Family history of a similar condition
- [ ] Gingivitis

13. Q10. Select 3 differential diagnoses *

*Check all that apply.*

- [ ] Generalized enamel abrasion
- [ ] Dentinogenesis imperfect
- [ ] Extrinsic discoloration
- [ ] Amelogenesis imperfecta
- [ ] Genetic pigmentation
- [ ] Calculus disposition
- [ ] Fluorosis
- [ ] Generalized attrition
- [ ] Enamel hypoplasia
- [ ] Enamel opacities
- [ ] Generalized enamel hypo mineralization

The following question deals with your ability to use the results of investigations to refine your diagnostic hypotheses.

use the Likert scale to indicate how likely this hypothesis becomes

14. Q11. if your diagnostic hypothesis was (Amelogenesis Imperfecta), and you find the result of investigation was (Generalized brownish discoloration with white patch and pitting of enamel), this hypothesis becomes: *

choose 1

*Mark only one oval.*

- -2= ruled out or almost ruled out
- -1=less probable
- 0=neither less nor more probable
- +1=more probable
- +2=certain or almost certain

15. Q12. if your diagnostic hypothesis was (External stain), and then you find the result of investigation was that (Staining of all tooth surfaces for incisors and first molars), this hypothesis becomes: *

*Mark only one oval.*

- -2= ruled out or almost ruled out
- -1=less probable
- 0=neither less nor more probable
- +1=more probable
- +2=certain or almost certain

16. Q13. if your diagnostic hypothesis was (Tooth fluorosis), and then you find that the result of investigation was (Marked wear), this hypothesis becomes: *

*Mark only one oval.*

- -2= ruled out or almost ruled out
- -1=less probable
- 0=neither less nor more probable
- +1=more probable
- +2=certain or almost certain

17. Q14. if your diagnostic hypothesis was (Congenital enamel defect), and then you find the result of investigation was (Open bite), this hypothesis becomes: *

*Mark only one oval.*

- -2= ruled out or almost ruled out
- -1=less probable
- 0=neither less nor more probable
- +1=more probable
- +2=certain or almost certain

18. Q15. if your diagnostic hypothesis was ( Tooth fluorosis), and then you find the result of investigation was (Used to drink ground water when he was in India), this hypothesis becomes: *

*Mark only one oval.*

- -2= ruled out or almost ruled out
- -1=less probable
- 0=neither less nor more probable
- +1=more probable
- +2=certain or almost certain

19. Q16. if your diagnostic hypothesis was (Amelogenesis imperfecta), and then you find the result of investigation was (Both mother and father have similar condition whereas young siblings have not), this hypothesis becomes: *

*Mark only one oval.*

- -2= ruled out or almost ruled out
- -1=less probable
- 0=neither less nor more probable
- +1=more probable
- +2=certain or almost certain

Case 3

A 53-year-old, non-smoking male came to your clinic complaining of bleeding gums and bad breath. He reported that he visits the dentist only when having toothache. He also reported that he brushes his teeth only once a day and does not use dental floss. He also wanted to replace his missing teeth.

20. Q17. At this stage, what do you think are the possible causes for his symptoms? (please provide two)

_______________________

Past medical history: In the review of symptoms he reported mild fatigue. He also reported that his father died of a heart attack at the age of 66 and his mother was alive and taking medication for diabetes mellitus. He reported recent weight loss, you noticed central obesity. Past dental history: The patient reported that in the past he had required many fillings and had a lot of teeth extracted. Intraoral examination: Examination reveals multiple missing teeth with several amalgam restorations. The gingiva demonstrated moderate to severe inflammation, being more pronounced in the papillae. Periodontal abscesses and moderate plaque accumulations were present. Probing depth ranged from 2 to 8 mm and the remaining molars have furcation involvements and variable degrees of mobility. This is the OPG of the patient

His OPG

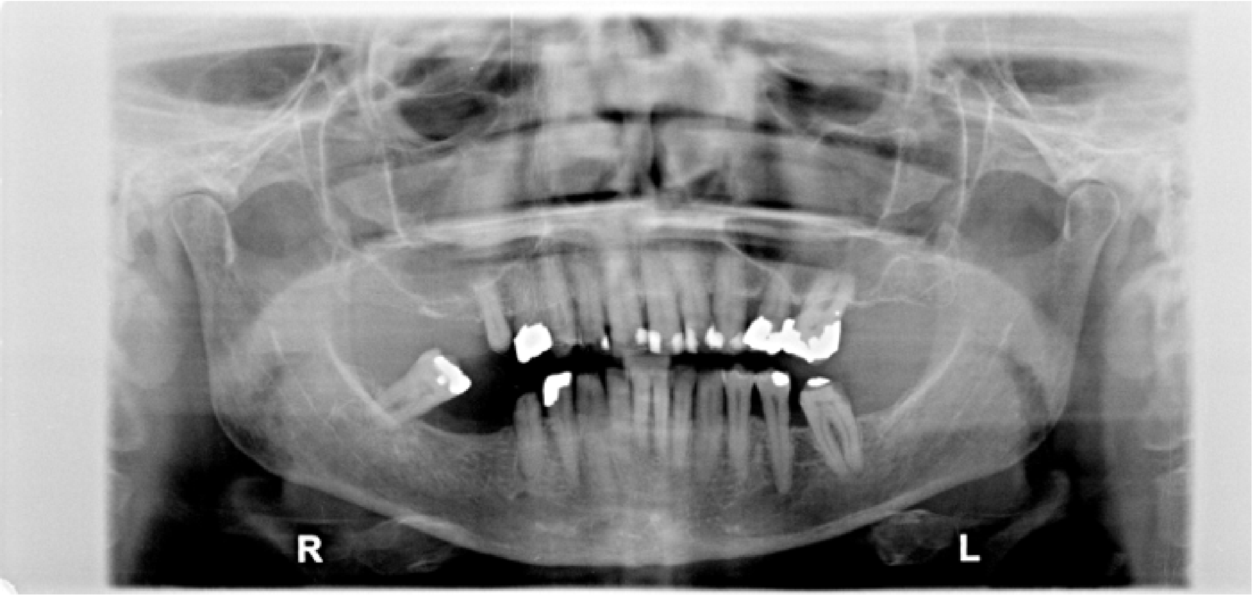

21. Q18. Based on the information provided, what is your most probable diagnosis of his periodontal problem?

22. Q19. Referring to the picture, please provide a suitable diagnosis to the radiolucency in the left angle of the mandible.

23. Q20. You offered endodontic treatment and crowns for multiple teeth, and then do a removable partial denture. However, the patient tells you that he cannot afford the cost of this comprehensive treatment. He suggests that you extract his remaining teeth and do a complete denture. You also need to do a complete denture in order to finish your course requirements. What is your decision in this case? *

24. Q21. A few weeks after giving oral hygiene instructions, scaling and root planning, and endodontic treatment for UR4 and LR5 you notice the oral hygiene has improved but tissue inflammation remains. You decide to refer the patient to a periodontist. The specialist performs surgical periodontal treatment in the maxillary right quadrant. The healing response is fair with persistence of inflammation of the gingival tissue even in the treated area. A few months later the patient underwent another surgical treatment to the maxillary left quadrant which was similar to the first surgical procedure. Unfortunately, the post surgical course was as before and both surgical treatments were unsuccessful with persistent inflammation. What do you think is the possible causes for his unimproved periodontal condition?

Case 4

A lady rushes into your emergency dental clinic with her 4 year old daughter who is crying and has blood drooling from her mouth.

25. Q22. What are the possible causes for her problem? state two

26. Q23. What investigations will help you to specify the possible causes? *

The mother reported that her daughter had accidentally fallen on her face and she took her child straight away to your clinic.

27. Q24. You conduct an intraoral examination and find that crowns of both upper primary central incisors are palatally displaced but quite firm in this position. Your most likely management will include: (choose 2) *

*Check all that apply.*

- [ ] • Extraction of both upper centrals, since they are deciduous
- [ ] • Trying to pull them back to their position
- [ ] • Leave them if not interfering with occlusion
- [ ] • Refer the patient to a maxillofacial surgeon
- [ ] • Take an x-ray

28. Q25. If these teeth had been intruded apically how would this change your management plan?

case 5

This is a picture of a seven-year old boy who came to your dental office with his parents. His dad was concerned with his son's front teeth which had the lesion shown in the photograph.

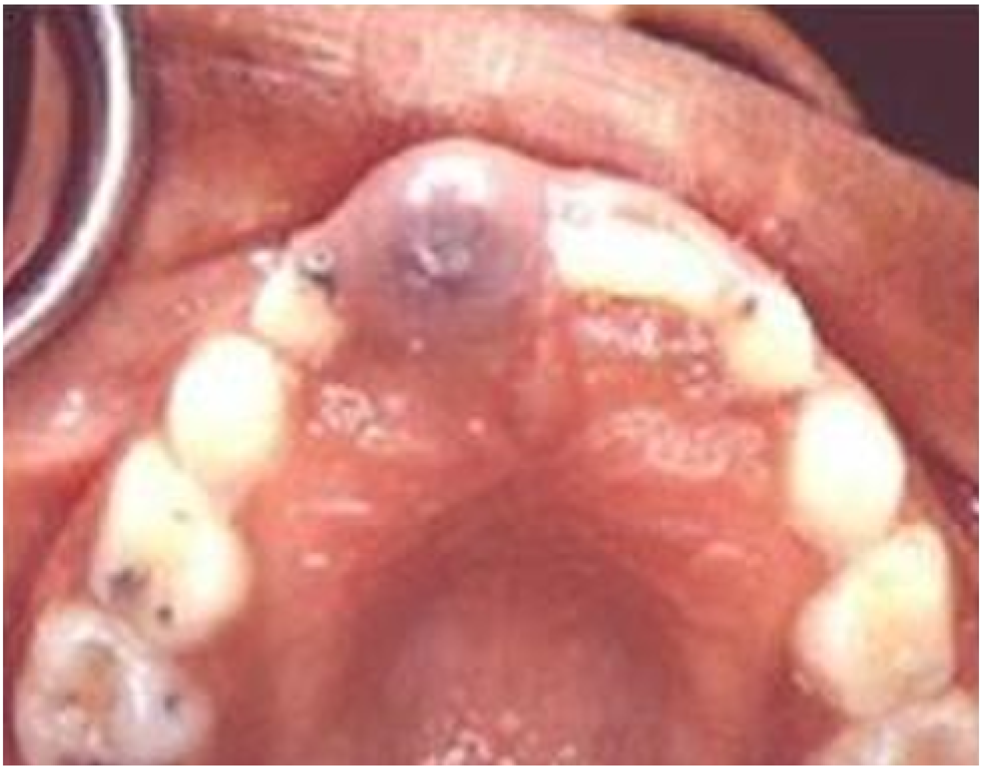

29. Q26. How would you describe this lesion?

30. Q27. What are the differential diagnoses of this lesion? list up to 2)

31. Q28. The following question deals with your ability to use the results of investigations to refine your diagnostic hypotheses. If you diagnostic hypothesis was (Localized gingival inflammation ), and the result of investigation was that (The patient was playing with a pencil in his mouth), this hypothesis becomes *

*Mark only one oval.*

- -2= ruled out or almost ruled out
- -1=less probable
- 0=neither less nor more probable
- +1=more probable
- +2=certain or almost certain

32. Q29. if your diagnostic hypothesis was (Genetic gingival pigmentation), and then you find the result of investigation was (The mother has got genetic pigmentation), this hypothesis becomes: *

*Mark only one oval.*

- -2= ruled out or almost ruled out
- -1=less probable
- 0=neither less nor more probable
- +1=more probable
- +2=certain or almost certain

33. Q30. if your diagnostic hypothesis was (Eruption cyst), and then you find the result of investigation was (This lesion is slightly painful upon palpation), this hypothesis becomes: *

*Mark only one oval.*

- -2= ruled out or almost ruled out
- -1=less probable
- 0=neither less nor more probable
- +1=more probable
- +2=certain or almost certain

34. Q31. if your diagnostic hypothesis was (Eruption haematoma), and then you find the result of investigation was (Periapical film of this patient), this hypothesis becomes: *

*Mark only one oval.*

- -2= ruled out or almost ruled out
- -1=less probable
- 0=neither less nor more probable
- +1=more probable
- +2=certain or almost certain

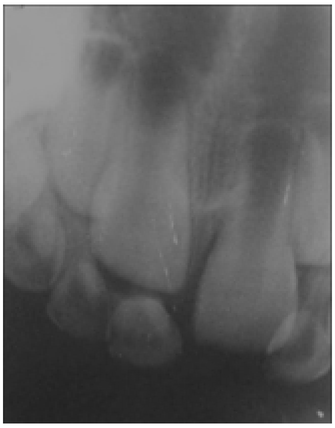

last section (self efficacy)

35. I can always manage to solve difficult problems if I try hard enough. *

*Mark only one oval.*

- 1 = Not at all true
- 2 = Hardly true
- 3 = Moderately true
- 4 = Exactly true

36. If someone opposes me, I can find the means and ways to get what I want. *

*Mark only one oval.*

- 1 = Not at all true
- 2 = Hardly true
- 3 = Moderately true
- 4 = Exactly true

37. It is easy for me to stick to my aims and accomplish my goals. *

*Mark only one oval.*

- 1 = Not at all true
- 2 = Hardly true
- 3 = Moderately true
- 4 = Exactly true

38. I am confident that I could deal efficiently with unexpected events. *

*Mark only one oval.*

- 1 = Not at all true
- 2 = Hardly true
- 3 = Moderately true
- 4 = Exactly true

39. Thanks to my resourcefulness, I know how to handle unforeseen situations. *

*Mark only one oval.*

- 1 = Not at all true
- 2 = Hardly true
- 3 = Moderately true
- 4 = Exactly true

40. I can solve most problems if I invest the necessary effort. *

*Mark only one oval.*

- 1 = Not at all true
- 2 = Hardly true
- 3 = Moderately true
- 4 = Exactly true

41. I can remain calm when facing difficulties because I can rely on my coping abilities. *

*Mark only one oval.*

- 1 = Not at all true
- 2 = Hardly true
- 3 = Moderately true
- 4 = Exactly true

42. When I am confronted with a problem, I can usually find several solutions. *

*Mark only one oval.*

- 1 = Not at all true
- 2 = Hardly true
- 3 = Moderately true
- 4 = Exactly true

43. If I am in trouble, I can usually think of a solution. *

*Mark only one oval.*

- 1 = Not at all true
- 2 = Hardly true
- 3 = Moderately true
- 4 = Exactly true

44. I can usually handle whatever comes my way. *

*Mark only one oval.*

- 1 = Not at all true
- 2 = Hardly true
- 3 = Moderately true
- 4 = Exactly true

45. Thank you for your contribution. Please enter your email address to take part in the raffle.

This content is neither created nor endorsed by Google.

Google Forms
