# Supplementary material for: Does Self-Efficacy Affect Clinical Reasoning in Dental Students?
Source: Int Dent J. 2022 Jun 23;72(6):872–8. doi: 10.1016/j.identj.2022.05.006 (PMC9676534; doi:10.1016/j.identj.2022.05.006)
Supplement: Supplementary file 2 [file mmc2.docx]

| Question Number | Question type | Total mark |
| --- | --- | --- |
| Q1 | Open-ended | 3 |
| Q2 | Open-ended | 1 |
| Q3 | Open-ended | 1 |
| Q4 | MCQ | 1 |
| Q5 | Open-ended | 1 |
| Q6 | Open-ended | 3 |
| Q7 | MCQ | 1 |
| Q8 | MCQ | 3 |
| Q9 | MCQ | 3 |
| Q10 | MCQ | 3 |
| Q11 | MCQ | 1 |
| Q12 | MCQ | 1 |
| Q13 | MCQ | 1 |
| Q14 | MCQ | 1 |
| Q15 | MCQ | 1 |
| Q16 | MCQ | 1 |
| Q17 | Open-ended | 2 |
| Q18 | Open-ended | 1 |
| Q19 | Open-ended | 1 |
| Q20 | Open-ended | 1 |
| Q21 | Open-ended | 1 |
| Q22 | Open-ended | 2 |
| Q23 | Open-ended | 1 |
| Q24 | MCQ | 2 |
| Q25 | Open-ended | 1 |
| Q26 | Open-ended | 1 |
| Q27 | Open-ended | 2 |
| Q28 | MCQ | 1 |
| Q29 | MCQ | 1 |
| Q30 | MCQ | 1 |
| Q31 | MCQ | 1 |

Marking of the Clinical Reasoning Test
