# Supplementary material for: Does Self-Efficacy Affect Clinical Reasoning in Dental Students?
Source: Int Dent J. 2022 Jun 23;72(6):872–8. doi: 10.1016/j.identj.2022.05.006 (PMC9676534; doi:10.1016/j.identj.2022.05.006)
Supplement: Supplementary file 3 [file mmc3.pdf]

Screen Shots for Clinical Reasoning Test

Clinical Reasoning Test

CASE 1

A 30-year-old lady came to your dental office complaining of pain related to her lower left molars, especially when biting. She repoted the pain as moderate.

Past medical and dental history:
the patient is medically fit and is not on any medication. She reported that she is used to having regular visits to the dentist. Her last visit to the dentist was about 3 weeks ago.

Extra-oral examination
No abnormality observed.

Intra-oral examination
Fairly good oral hygiene was noticed with healthy gingival tissue. She has class I occlusion with cross bite in her canines on both sides.Class I occlusal composite restorations to her teeth: UR7, UL6, LL7 and LR7. The UR6 has a class I amalgam filling. She also has fissure sealant for her premolars. The LL7, with an adequate occlusal class I composite, was sensitive to vertical percussion. Generalised attrition to occusal surfaces was observed. By asking the patient, she confirmed that she often clenches her teeth especially when stressed.

Radiographic examination
No periapical lesion was noticed in the area of concern.

3. List the features from the case description above that could help you in making a diagnosis for her complaint (List at least three)

1

2

3

4. At this stage, what is your most likely diagnosis for the LL7?

5. What further investigations will help you to diagnose this problem, or confirm your diagnosis?

Clinical Reasoning Test

6. If you suppose that this lady has a high composite filling for LL7, and by reviewing her dental record, it was found that her dentist had used composite for anterior teeth. How can you manage her problem? Choose the best answer.

- Slightly reduce the filling by 0.5mm
- Replace the filling by a new one with composite for posterior teeth
- Do a night gaurd (splint)
- Ignore the problem as it will go away with time, it will undergo attrition by clenching.
- Identify high points and reduce the filling
- Replace the filling with amalgam

7. How could this problem be avoided in the first place?

Clinical Reasoning Test

CASE 2

A 16 year old male came to your clinic complaining of brownish discolouration and irregular teeth related to his anterior teeth as seen in the picture. He is shy and does not want to show his teeth. He also stated that he's had bad teeth since he was a child but he's now started to take care of them and they are improving. His nationality is Indian and he came to live in the UK 8 years ago.

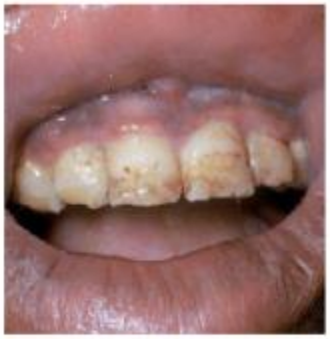

8. What clinical features would you focus on in making your diagnosis for this patient? List up to 3

1 ____________________

2 ____________________

3 ____________________

9. If you could choose the course of action to follow, what would you like to do immediately? (choose one)

- ○ I don't know
- ○ Ask the patient more questions
- ○ Conduct intraoral examination
- ○ Give him oral hygiene instructions and do scaling.
- ○ Take an x-ray
- ○ Refer the patient to an oral pathology specialist

Clinical Reasoning Test

10. From the list below select the (THREE) most important questions that you would like to ask the patient.

- ☐ Do you have other siblings affected?
- ☐ How frequently do you brush your teeth?
- ☐ Do you use fluoridated tooth paste?
- ☐ Do you have any pain or sensitivity?
- ☐ Have you experienced trauma to your teeth?
- ☐ Do you drink lots of coffee or tea?
- ☐ What type of water did you used to drink when you were younger?
- ☐ Did you have any serious illness when you were a baby?
- ☐ Do you use Chlorhexidine mouth wash?

Clinical Reasoning Test

The patient's answers to the previous questions are:
1.How frequently do you brush your teeth? I brush my teeth twice a day.
2.Did you have any serious illness when you were a baby? No I don't think so.
3.Do you use Chlorhexidine mouth wash? Yes I usually use it once a day for about three months.
4.What type of water did you used to drink when you were younger? Tap water.
5.Do you have any pain or sensitivity? No
6.Do you drink lots of coffee or tea? I usually take two cups of black coffee daily.
7.Do you use fluoridated tooth paste? Yes
8.Do you have other siblings affected? I have a 4-year old sister and she hasn't got this problem.
9.Have you experienced trauma to your teeth? Can't remember.

11. Select (THREE) results from the list below that you would expect to find during examination:

- ☐ Generalised discolouration
- ☐ Caries
- ☐ Generalised recession
- ☐ Localised discolouration
- ☐ Open bite
- ☐ Small teeth
- ☐ Loss of proximal contact
- ☐ Abrasion and horizontal bone loss
- ☐ Vertical bone loss
- ☐ Submandibular gland tenderness
- ☐ Family history of a similar condition
- ☐ Gingivitis

12. Choose (THREE) differential diagnoses

- ☐ Generalised enamel abrasion
- ☐ Dentinogenesis imperfecta
- ☐ Extrinsic discolouration
- ☐ Amelogenesis imperfecta
- ☐ Genetic pigmentation
- ☐ Calculus deposition
- ☐ Fluorosis
- ☐ Generalised attrition
- ☐ Enamel hypoplasia
- ☐ Enamel opacities
- ☐ Generalised enamel hypomineralisation

13. The following questions deal with your ability to use the results of investigations to refine your diagnostic hypotheses

	Use the Likert scale to indicate how likely this hypothesis becomes
If your diagnostic hypothesis was (Amelogenesis imperfecta), and you find the result of investigation was (generalised brownish discolouration with white patch and pitting of enamel), this hypothesis becomes:	[▾]

14. Use the Likert scale to indicate how likely this hypothesis becomes

If your diagnostic hypothesis was (external stain), and then you find the result of investigation was that (staining of all tooth surfaces for incisors and first molars), this hypothesis becomes:	[▾]

15. Use the Likert scale to indicate how likely this hypothesis becomes

If your diagnostic hypothesis was (tooth fluorosis) and you find the result of investigation was (marked wear), this hypothesis becomes?	[▾]

Clinical Reasoning Test

16. Use the Likert scale to indicate how likely this hypothesis becomes

If your diagnostic hypothesis was (congenital enamel defect), and then you find the result of investigation was (open bite), this hypothesis becomes?

17. Use the Likert scale to indicate how likely this hypothesis becomes

If your diagnostic hypothesis was (tooth fluorosis), and you find the result of investigation was that (the patient had used to drink ground water when he was in India), this hypothesis becomes:

18. Use the Likert scale to indicate how likely this hypothesis becomes

If your diagnostic hypothesis was (Amelogenesis imperfecta), and you find the result of investigation was that (both mother and father have similar condition whereas young siblings have not), this hypothesis becomes:

Clinical Reasoning Test

CASE 3

A 53-year-old, non-smoking male came to your clinic complaining of bleeding gums and bad breath. He reported that he visits the dentist only when having toothache. He also reported that he brushes his teeth only once a day and does not use dental floss. He also wanted to replace his missing teeth.

19. At this stage, what do you think are the possible causes for his symptoms (please provide TWO causes)

1

2

(Past medical history)
In the review of symptoms he reported mild fatigue. He also reported that his father died of a heart attack at the age of 66 and his mother was alive and taking medication for diabetes mellitus. He reported recent weight loss, but you notice central obesity.
(Past dental history)
The patient reported that in the past he had required many fillings and had a lot of teeth extracted.
(Intra oral examination)
Examination reveals multiple missing teeth with several amalgam restorations. The gingiva demonstrated moderate to severe inflammation, being more pronounced in the papillae. Periodontal abscesses and moderate plaque accumulations were present. Probing depth ranged from 2 to 8 mm and the remaining molars have furcation involvements and variable degrees of mobility.
This is the OPG of the patient

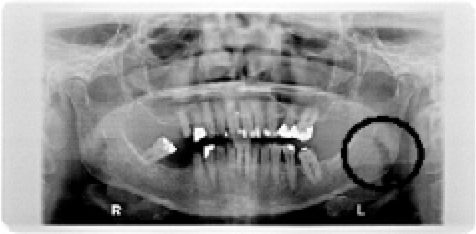

20. Based on the information provided, what is your most probable diagnosis of his periodontal problem?

21. Referring to the picture, please provide a suitable diagnosis to the radiopacity in the left angle of the mandible.

22. You offer endodontic treatment and crowns for multiple teeth, and then do a removable partial denture. However, the patient tells you that he cannot afford the cost of this comprehensive treatment. He suggests that you extract his remaining teeth and do a complete denture. You also need to do a complete denture in order to finish your course requirements. What can you do in this case?

A few weeks after giving oral hygiene instructions, scaling and root planing, and endodontic treatment for UR4 and LR5 you notice the oral hygiene has improved but tissue inflammation remains. You decide to refer the patient to a periodontist. The specialist performs surgical periodontal treatment in the maxillary right quadrant. The healing response is fair with persistence of inflammation of the gingival tissue even in the treated area. A few months later the patient underwent another surgical treatment to the maxillary left quadrant which was similar to the first surgical procedure. Unfortunately, the post surgical course was as before and both surgical treatments were unsuccessful with persistant inflammation.

23. What do you think is the possible cause of the unimproved periodontal condition?

Clinical Reasoning Test

CASE 4

24. A lady rushes into your emergency dental clinic with her 4-year old daughter who is crying and has blood drooling from her mouth.
What are the possible causes for her problem?

1.
2.

25. What investigations will help you to specify the possible causes?

The mother reported that her daughter had accidentally fallen on her face and she took her child straight away to your clinic.

26. You conduct an intraoral examination and find that crowns of both upper primary central incisors are palatally displaced but quite firm in this position.
Your most likely management will include: (CHOOSE TWO)

- ☐ - Extraction of both upper centrals, since they are deciduous
- ☐ - Trying to pull them back to their position
- ☐ - Leave them if not interfering with occlusion
- ☐ - Refer the patient to a maxillofacial surgeon
- ☐ - Take an x-ray

27. If these teeth had been intruded apically how would this change your management plan?

Clinical Reasoning Test

CASE 5

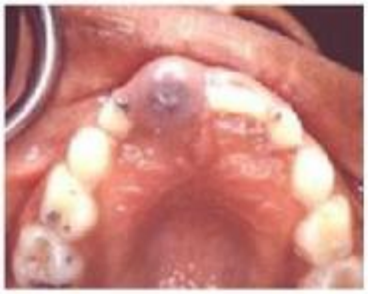

This is a picture of a seven-year old boy who came to your dental office with his parents. His dad was concerned with his son's front teeth which had the lesion shown in the photograph.

28. How would you describe this lesion?

29. What are the differential diagnoses of this lesion? (LIST UP TO TWO)

1

2

30. The following questions deal with your ability to use the results of investigations to refine your diagnostic hypotheses. Use the Likert scale to indicate how likely this hypothesis becomes.

If your diagnostic hypothesis was (localised gingival inflammation), and then you find the result of investigation was that (the patient was playing with a pencil in his mouth), this hypothesis becomes:

31. Use the Likert scale to indicate how likely this hypothesis becomes

If your diagnostic hypothesis was (genetic gingival pigmentation), and then you find the result of investigation was that(the mother has got genetic pigmentation), this hypothesis becomes:

Clinical Reasoning Test

32. Use the Likert scale to indicate how likely this hypothesis becomes

If your diagnostic hypothesis was (eruption cyst), and then you find the result of investigation was that(this lesion is slightly painful upon palpation), this hypothesis becomes:

33. Use the Likert scale to indicate how likely this hypothesis becomes

If your diagnostic hypothesis was (euption haematoma), and you see the periapical film of the patient below, this hypothesis becomes:

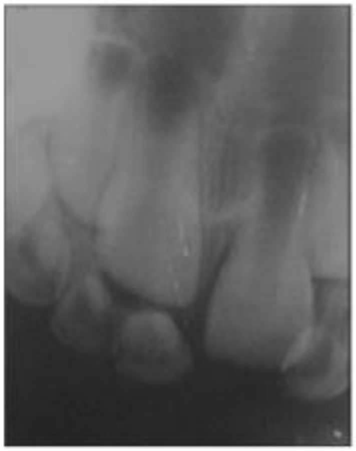

34. Please enter your email address to take part in the raffle.

35. We would like to conduct qualitative research about clinical reasoning using interviews. We would like you to volunteer for our qualitative research. If you are a Birmingham dental student and happy to be interviewed(less than 20 minutes), could you please provide your email address below. Compansation for your time will be provided for you

Clinical reasoning test marking

Question Number	Question type	Total mark
Q1	Open-ended	3
Q2	Open-ended	1
Q3	Open-ended	1
Q4	MCQ	1
Q5	Open-ended	1
Q6	Open-ended	3
Q7	MCQ	1
Q8	MCQ	3
Q9	MCQ	3
Q10	MCQ	3
Q11	MCQ	1
Q12	MCQ	1
Q13	MCQ	1
Q14	MCQ	1
Q15	MCQ	1
Q16	MCQ	1
Q17	Open-ended	2
Q18	Open-ended	1
Q19	Open-ended	1
Q20	Open-ended	1
Q21	Open-ended	1
Q22	Open-ended	2
Q23	Open-ended	1
Q24	MCQ	2
Q25	Open-ended	1
Q26	Open-ended	1
Q27	Open-ended	2
Q28	MCQ	1
Q29	MCQ	1
Q30	MCQ	1
Q31	MCQ	1
