# Supplementary material for: Does Self-Efficacy Affect Clinical Reasoning in Dental Students?
Source: Int Dent J. 2022 Jun 23;72(6):872–8. doi: 10.1016/j.identj.2022.05.006 (PMC9676534; doi:10.1016/j.identj.2022.05.006)
Supplement: Supplementary file 4 [file mmc4.pdf]

ELSEVIER

Certificate of Elsevier Language Editing Services

The following article was edited by Elsevier Language Editing Services:

"Dental students' educational achievement in relation to their clinical reasoning and self-efficacy"

Authored by:

Ebtihaj Nafea

Date: 01-Apr-2022

Serial number: LE-236202-36CD0D752A0B
